# A novel abnormality annotation database for COVID-19 affected frontal lung X-rays

**DOI:** 10.1371/journal.pone.0271931

**Published:** 2022-10-14

**Authors:** Surbhi Mittal, Vasantha Kumar Venugopal, Vikash Kumar Agarwal, Manu Malhotra, Jagneet Singh Chatha, Savinay Kapur, Ankur Gupta, Vikas Batra, Puspita Majumdar, Aakarsh Malhotra, Kartik Thakral, Saheb Chhabra, Mayank Vatsa, Richa Singh, Santanu Chaudhury

**Affiliations:** 1 Department of Computer Science, IIT Jodhpur, Karwar, Rajasthan, India; 2 Mahajan Imaging, New Delhi, India; 3 Department of Computer Science, IIIT Delhi, New Delhi, India; Fuzhou University, CHINA

## Abstract

Consistent clinical observations of characteristic findings of COVID-19 pneumonia on chest X-rays have attracted the research community to strive to provide a fast and reliable method for screening suspected patients. Several machine learning algorithms have been proposed to find the abnormalities in the lungs using chest X-rays specific to COVID-19 pneumonia and distinguish them from other etiologies of pneumonia. However, despite the enormous magnitude of the pandemic, there are very few instances of public databases of COVID-19 pneumonia, and to the best of our knowledge, there is no database with annotation of abnormalities on the chest X-rays of COVID-19 affected patients. Annotated databases of X-rays can be of significant value in the design and development of algorithms for disease prediction. Further, explainability analysis for the performance of existing or new deep learning algorithms will be enhanced significantly with access to ground-truth abnormality annotations. The proposed COVID Abnormality Annotation for X-Rays (CAAXR) database is built upon the BIMCV-COVID19+ database which is a large-scale dataset containing COVID-19+ chest X-rays. The primary contribution of this study is the annotation of the abnormalities in over 1700 frontal chest X-rays. Further, we define protocols for semantic segmentation as well as classification for robust evaluation of algorithms. We provide benchmark results on the defined protocols using popular deep learning models such as DenseNet, ResNet, MobileNet, and VGG for classification, and UNet, SegNet, and Mask-RCNN for semantic segmentation. The classwise accuracy, sensitivity, and AUC-ROC scores are reported for the classification models, and the IoU and DICE scores are reported for the segmentation models.

## Introduction

**Fig 1:**
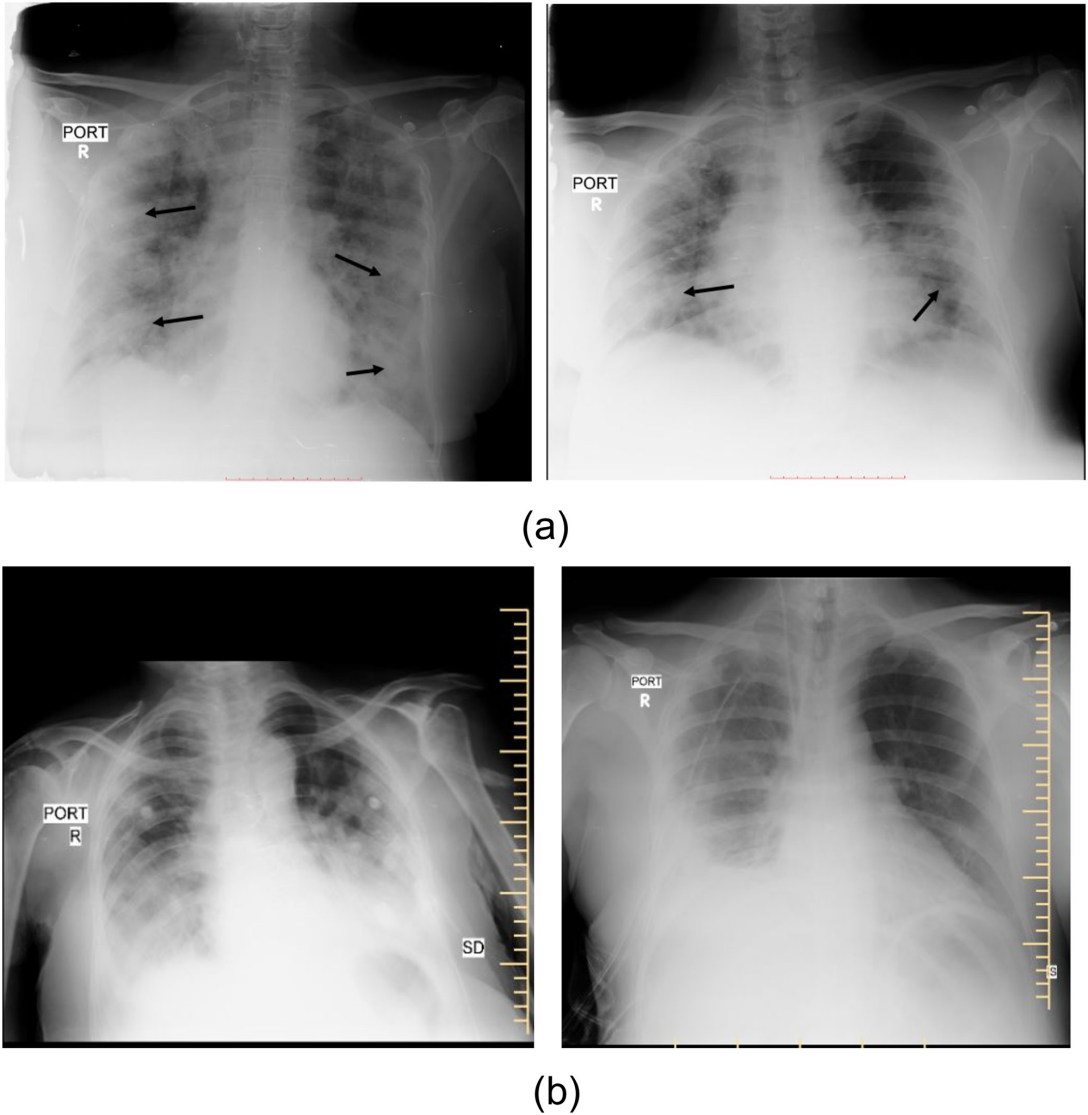
Chest X-rays of (a) RT-PCR proven COVID-19 pneumonia in cases showing the typical bilateral peripheral consolidations and ground-glass opacities. (b) Non-COVID-19 pneumonia in cases showing the lobar distribution of consolidations with pleural effusion.

In the face of the SARS-CoV2 or Coronavirus pandemic, the lifestyle and health of a large number of people are adversely hit. More than 500 million people have been reportedly exposed to the COVID-19 virus, globally [1]. With the easy transmission of the virus, mass screening for symptoms and testing of suspected patients has become necessary. Studies have shown that radiological investigations such as chest X-rays and CT scans can be employed in the diagnostic workflow of COVID-19 patients [2, 3]. Even though CT scans are more sensitive and specific for the diagnosis of COVID-19 pneumonia, X-rays remain popular due to their lower cost, wide availability, mobility, and ease of disinfection. COVID-19 pneumonia has been shown to have a characteristic spectrum of findings on chest X-rays [4, 5]. These are bilateral peripheral patchy opacities in predominant lower lobe distributions [4, 5]. Lymphadenopathy and pleural effusions are unusual and their presence in patients with COVID-19 indicates secondary infection or other underlying pathologies. While there is a possibility of overlap of these findings with viral or other atypical pneumonia [6], they are distinctively different from the more common typical pneumonia of bacterial origin. These differences offer an opportunity to use X-rays for screening and diagnosis of COVID-19 pneumonia, differentiating them from typical pneumonia. Consolidation is the pathognomonic finding in both forms of pneumonia [7]. Some of these differences can be observed in Fig 1. For differentiating COVID-19 pneumonia, frontal chest X-rays (CXR) remain as a popular choice [8–17]. These studies utilize the existing databases of COVID-19 affected CXR, including BIMCV-COVID19+ [18], COVID-19 Image Data Collection on GitHub [19], COVIDx database [15], and others [20–24]. Most of these datasets are limited in numbers and also lack abnormality localization information. Other CXR datasets have been discussed in detail in the Related Work section.

Annotating abnormalities and findings can prove to be a useful source of supervision in training ML and DL models. It is especially imperative in the medical domain that diagnoses made by the model can be explained before any further action is undertaken. With this motivation, in this work, we build upon the BIMCV-COVID19+ database which is a large-scale database containing more than 2,000 chest X-ray images. We provide abnormality localization of frontal chest X-rays and introduce the COVID Abnormality Annotation for X-Rays (CAAXR) database. The marked annotations are performed by six experienced radiologists. The annotations include a comprehensive set of abnormalities, including but not limited to atelectasis, consolidation, pleural effusion, and edema. Non-lung abnormalities but visible on the CXR like a fracture, cardiomegaly, and presence of support devices are also noted. On 1749 different X-rays from the BIMCV-COVID19+ database, we mark a total of 3943 abnormalities as bounding boxes. This large-scale data can be used for experimentation of machine learning and deep learning algorithms and can further be used for learning radiological patterns observed in COVID-19 patients.

We further propose a comprehensive set of protocols for the robust evaluation of machine learning and deep learning algorithms. A common problem in the evaluation of COVID-19 pneumonia classifiers has been the lack of non-definition of validation framework and non-availability of common testing sets. This issue is further complicated as different algorithms have hugely diverging problem statements. Some of them classify X-rays into two classes (COVID, non-COVID pneumonia), some into three classes (COVID pneumonia, Non-COVID pneumonia, and normal) while a few classify them into four or more classes (COVID pneumonia, Non-COVID pneumonia, other diseases, and normal). To address this limitation and facilitate the usage of the BIMCV-COVID19+ database in conjunction with the proposed annotation, our study presents a robust evaluation mechanism. Using the proposed annotations, the designed protocols provide a four-way testing mechanism on a consolidated set of X-rays. The protocols include one protocol for semantic segmentation for abnormality localization, and a protocol each for two, three, and four class classification of X-rays. The segregation is based on COVID-19 pneumonia, non-COVID pneumonia, other abnormalities, and normal X-rays. The protocol also incorporates cross-validation, allowing algorithm developers to check the stability of their proposed algorithms. Lastly, we establish the baseline cross-validated performance for popular deep learning algorithms for each of the protocols. The performance is reported through IoU and DICE scores for segmentation, and through accuracy, sensitivity, and AUC-ROC scores for classification protocols. The protocols and annotation data will facilitate machine learning practitioners for robust and unified large-scale evaluation of their algorithms. By making these data and protocols available to the scientific community, we aim to advance the usage of X-rays as a viable solution for efficient COVID-19 screening.

The contributions of this paper are summarized below-

- We propose the COVID Abnormality Annotation for X-Rays (CAAXR) database which provides abnormality annotations for over 1700 COVID-19 infected chest X-rays. The annotations include bounding boxes for infected regions as well as the name of the radiological abnormality.
- We propose four classification and segmentation protocols for the robust evaluation of deep learning models.
- We provide detailed benchmark results using the defined classification and segmentation protocols for 7 popular deep learning models. The performance of these models is discussed based on a variety of evaluation metrics.

## Related work

The related literature for chest X-ray datasets containing abnormality annotations is limited. The widely used Chest-xray14 [25] dataset provides bounding box annotations for 983 CXR images. The annotations corresponding to abnormalities in the Chest-xray14 dataset are generated using automatic labels on medical reports. In comparison, the CheXpert dataset [26] uses radiologist-labeled validation and test sets, leading to a strong reference standard when compared to other datasets. It also enables robust evaluation of algorithms. Keeping these advantages in mind, we opted for the CheXpert dataset for selection of Non-COVID samples. The CheXplanation dataset [27] is a new annotated subset of the original CheXpert dataset. It is an abnormality annotated validation set consisting of 234 chest X-rays from 200 patients randomly sampled from the full dataset. The test set contains annotations for 668 X-rays but these annotations are only available for the corresponding CheXplanation competition.

All aforementioned datasets contain non-COVID chest X-rays. A limited number of datasets are available for COVID-19 CXRs including the COVID-19 Image Data Collection on GitHub [19] and CovidX database [15]. Both these datasets contain COVID-positive images assorted from various different sources. In fact, CovidX subsumes the Image Data Collection on GitHub database. As per the original paper, the CovidX dataset contained only 358 CXR images from 266 COVID-19 patient cases. Other databases include the Combined COVID-19 X-ray datasets [28] which includes 134 COVID-19 chest X-ray images. Synthetic datasets containing COVID samples have also been proposed in the literature [29–31]. Recent datasets include the BrixIA [32] and CPCXR [33] datasets. The different datasets have been summarized in Table 1. As evident from the table, most datasets constitute a small number of COVID-19 CXRs. On the other hand, the BIMCV-COVID19+ dataset [34] is a large-scale dataset with over 2000 X-rays of positive patients which led us to choose the BIMCV-COVID19+ dataset for annotation and subsequent protocol design. To the best of our knowledge, no existing datasets contain abnormality annotations by radiologists for COVID-19 infected CXRs.

**Table 1:** Summary of existing datasets containing COVID-19 positive chest X-rays.

Dataset	COVID-19 CXR Samples	Disease Region Annotation	Description
COVID-19 Image Data Collection [19]	11	✘	The COVID-19 Image Data Collection dataset was the first effort towards COVID-19 CXR data collection.
COVIDx [15]	385	✘	The COVIDx dataset combines a wide variety of infected chest CXRs. However, the number of COVID-19 CXRs is extremely limited.
COVID-19 Posteroanterior Chest X-Ray fused [33]	153	✘	The CPCXR dataset is a combination of three publicly available datasets.
BrixIA Covid-19 [32]	192	✘	The CXRs in the BrixIA dataset are annotated as per the BrixIA score which is a score to measure the degree of lung compromise.
Novel COVID-19 Chestxray Repository [30]	752	✘	The Novel COVID-19 Chestxray Repository dataset is a combination of CXRs collected from various sources including healthy and pneumonia-infected CXRs.
COVID-19 Radiography Database [35, 36]	3616	✘	The COVID-19 Radiography Database dataset is a combination of different datasets and collected as a part of a Kaggle competition with CXRs added incrementally.
BIMCV-COVID19+ [34]	2429	✘	The BIMCV dataset contains COVID-infected CXRs collected in Spain with a large number of infected CXRs.
**CAAXR (Ours)**	**1749**	✔	The proposed CAAXR dataset uses a subset of BIMCV-COVID19+ dataset and provides additional disease region annotations, unavailable\\in existing datasets.

## CAAXR dataset creation

This section describes the methodology for producing data and performing subsequent experimentation as a part of this study. The frontal chest X-rays (CXR) are drawn from existing public datasets, details of which are described below.

### Data sources

#### BIMCV-COVID19+ [18] dataset

BIMCV-COVID19+ dataset is a large dataset of chest X-rays and CT images of COVID-19 positive patients. It contains 1311 subjects and 2429 image studies. The dataset contains multiple studies of the same subject and multiple images per study in a few cases. Additionally, the dataset has phenotypic data and metadata information for acquired images. The metadata contains information such as study date, patients’ sex, the modality of study, and other details. The anonymized dataset also provides medical testing information of patients with regards to PCR, IGM, or other diagnostic tests along with the date of testing. This study focuses only on frontal chest radiograph images from the dataset. More details about the BIMCV-COVID19+ can be found in the preprint released with the dataset [34].

#### CheXpert [26] dataset

CheXpert dataset is a large chest X-ray dataset containing 223,414 images. It is a popular dataset for performing automatic CXR interpretation, containing radiologist-labeled data. Based on the radiological findings, each X-ray image is labeled either *0* (negative), *1* (positive), or *‘u’* (uncertain) for 14 pre-defined classes. The 14 classes are *Atelectasis*, *Cardiomegaly*, *Consolidation*, *Edema*, *Pleural Effusion*, *Pneumonia*, *Pneumothorax*, *Enlarged Cardiom.*, *Lung Lesion*, *Lung Opacity*, *Pleural Other*, *Fracture*, *Support Devices*, and, *No Finding*. Since the presence of the 14 observations in radiology reports is detected using an automatic labeler, there is a correlation among images belonging to different classes such as *Pneumonia* and *Consolidation*, and *Enlarged Cardiom.* and *Cardiomegaly*. According to [37], all instances in the CheXpert dataset are anonymized. For this study, a subset of fixed 7,212 images are acquired from the CheXpert dataset. Since the BIMCV-COVID19+ dataset contains only COVID-positive samples, we select healthy samples from the CheXpert database. Moreover, we select a large number of pneumonia unhealthy samples from the CheXpert dataset since COVID and pneumonia are closely related pathologies. While COVID-negative samples are provided at https://bimcv.cipf.es/bimcv-projects/bimcv-covid19/#1590859488150-148be708-c3f3, no related preprint has been released at the time of writing this paper.

### Data annotation

A total of 1,749 chest X-Rays have been annotated by six radiologists. The images were randomly assigned to different radiologists. All the six radiologists had more than 5 years experience, with two among them having 10 and 15 years of experience of reading chest X-rays. All the X-rays were annotated only once by a single radiologist. X-rays with doubtful findings were discussed, and consensus resolution was obtained by the independent effort of the annotator. An ideal process would have been annotations of each X-ray by multiple radiologists for consensus ground truth. However, due to the limited time and the urgent need to release data for meaningful use during the pandemic, the aforementioned process could not be followed.

**Fig 2:**
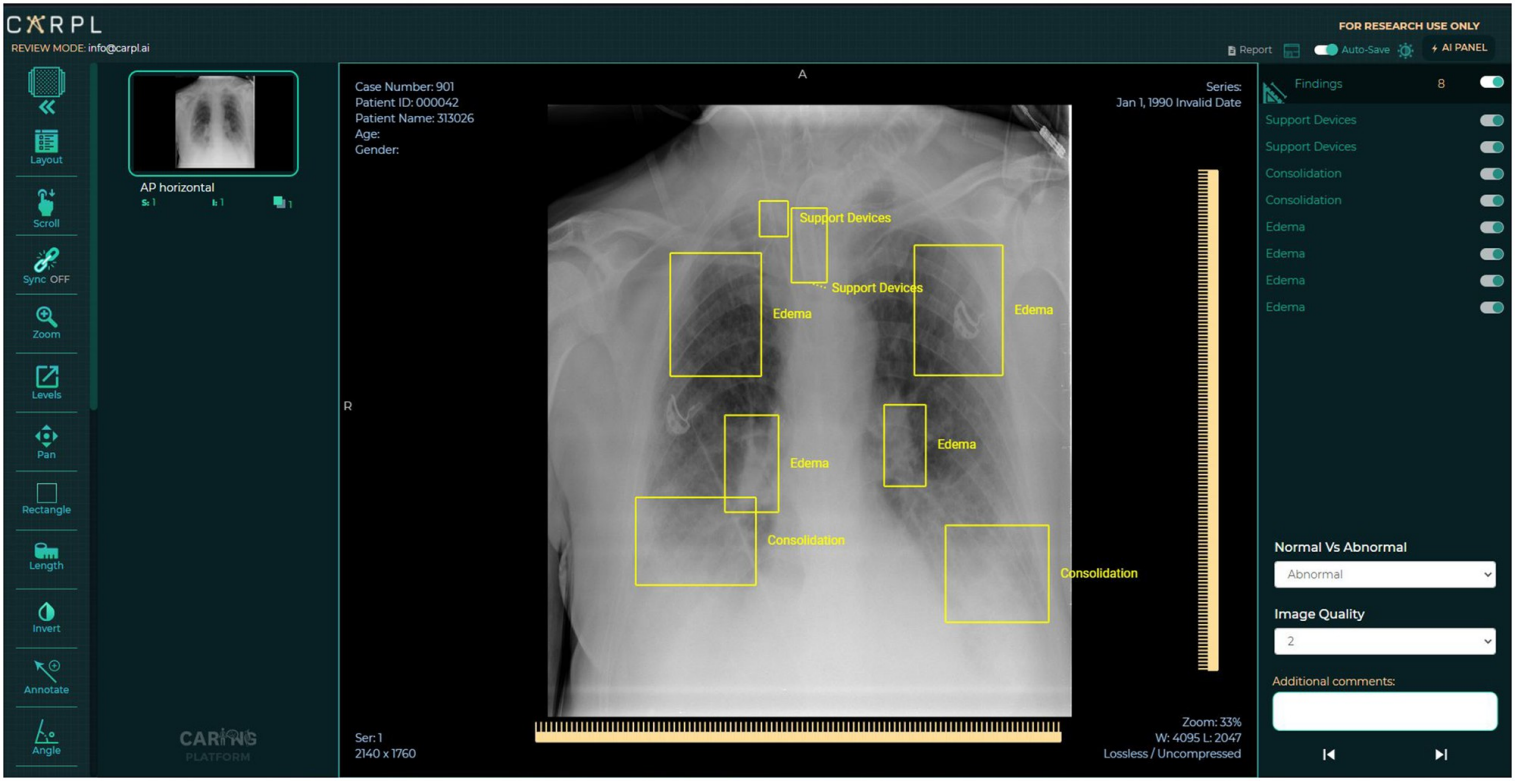
Representative image of the CARPL Annotation Platform tool used for loading DICOM files and subsequently for drawing bounding boxes around each image.

The CARPL Annotation Platform (CARING, New Delhi, INDIA) was used for annotation. It has an annotation management component based on Python and JavaScript and a viewer component based on ReactJS. In the presence of any abnormality as listed in the definition of the abnormality, the annotators used a bounding box annotation tool on the CARPL viewer to mark the pathological condition on the x-ray. In addition, the location of the support devices like pacemaker, valves and ICD tubes were also marked by bounding box annotations. Fig 2 shows a representative image of the annotation tool and the process. The X-rays were viewed serially by the radiologists for evaluation of whether they were normal or had any clinically significant findings. The definition of normalcy for the annotation was defined as follows:

1. Absence of the following pathological conditions: atelectasis, cardiomegaly, consolidation, consolidation/GGO, edema, enlarged cardio-mediastinum, fracture, hilar prominence, mediastinal mass, nodules, pleural effusion, other pleural pathologies, pneumothorax, and pulmonary edema.
2. Absence of any other finding outside the throacic cavity. This includes findings that the radiologist would have reported in their usual clinical practice as an abnormality needing intervention.

The annotations are created in the form of rectangular bounding boxes and have associated finding labels. The images are downloaded from the BIMCV-COVID19+ dataset [18] in 16-bit PNG format, converted to DICOM using python modules—Simple-ITK & NumPy. A JSON file was also provided in the BIMCV-COVID19+ dataset with every image having metadata of the DICOM file. The tags were added to the converted DICOM files using the python module PyDicom. The subsequently produced DICOM files are loaded onto the CARPL Annotation Platform (CARING, New Delhi, INDIA) and accessed by the radiologists who drew bounding boxes around each image (See Fig 2).

**Fig 3:**
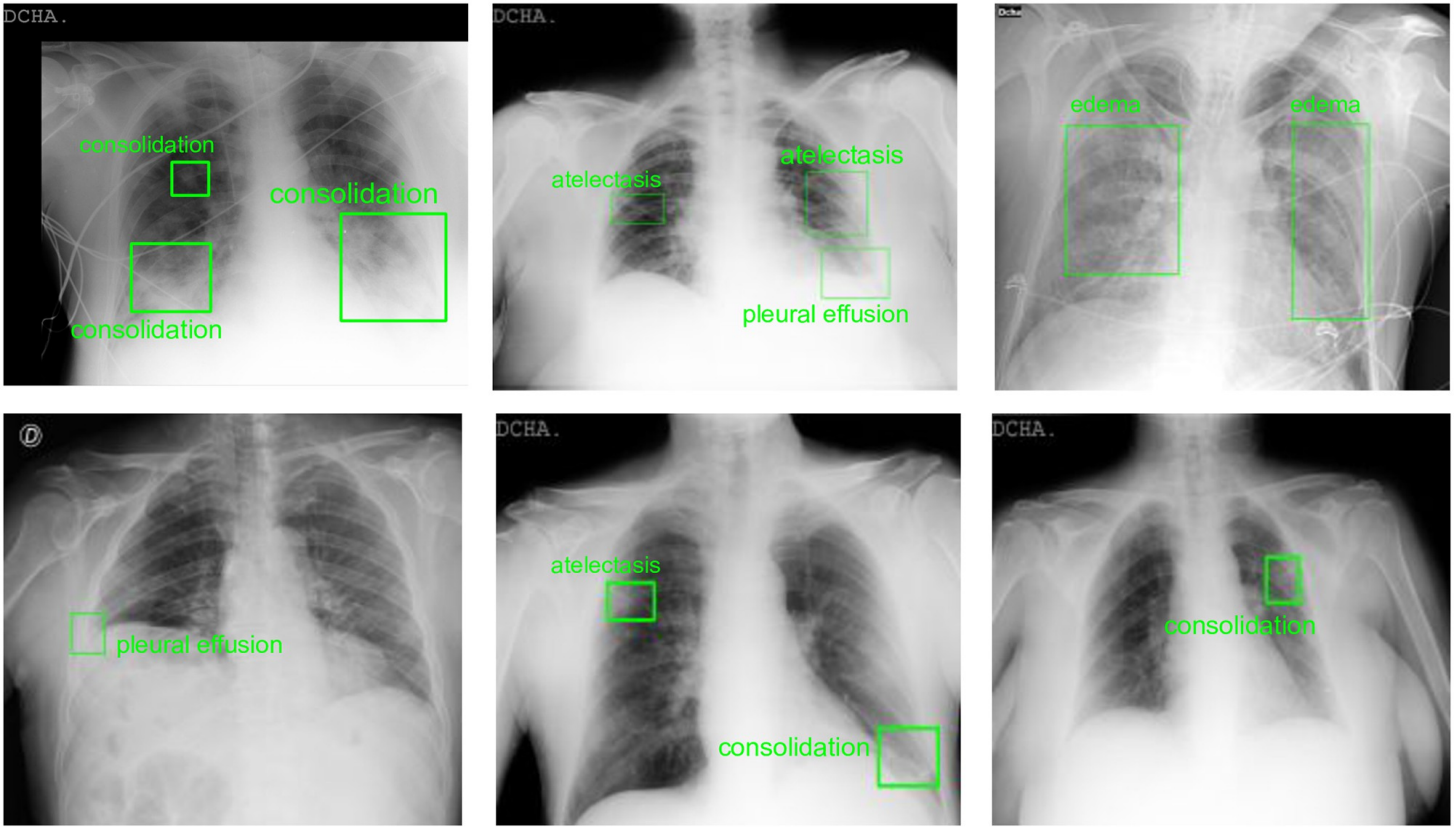
Samples of abnormality annotations performed for BIMCV-COVID19+ dataset. The regions annotated by the radiologists are depicted using green bounding boxes along with the abnormality annotation.

The pathologies that were marked on the image by bounding box include atelectasis, cardiomegaly, consolidation, consolidation/GGO, edema, enlarged cardio-mediastinum, fracture, hilar prominence, mediastinal mass, nodules, pleural effusion, pleural other, pneumothorax, and pulmonary edema. In addition, the location of the support devices like pacemakers, valves and ICD tubes were also marked by bounding box annotations. At the study level, the images are annotated for ‘Normal/Abnormal’ classification and for the image quality on a scale of ‘1-3’ (1- poor, 2- acceptable, and 3- good quality). Some samples for bounding box annotations can be seen in Fig 3. The annotations in the CAAXR dataset can be accessed at https://osf.io/b35xu/.

### Dataset documentation and availability

The data is available for access at two weblinks- http://iab-rubric.org/caaxr2020 and https://osf.io/b35xu/. The annotations and associated finding labels for CXR images are provided in ‘Bounding Boxes.csv’. The files can be downloaded directly from the aforementioned links and are only available for non-commercial use. The CSV file contains image filenames along with rectangular bounding box coordinates and related radiological findings. Each row corresponds to one bounding box annotation, and one CXR image may have multiple such annotations. The annotations for ‘Normal/Abnormal’ and image quality corresponding to CXR images are provided in ‘Normal_Abnormal.csv’. Each row in the CSV file specifies the image filename, its class (normal/abnormal), and the quality of the CXR image. The details of the proposed protocols in experimental design are also provided in the form of CSV files. Its details are as follows:

- There are two folders, namely- ‘train_val’ and ‘test’. The folders contain information regarding files used for training (training and validation) and testing, respectively.
- The ‘train_val’ folder contains four files, one for each protocol introduced. The files are named as ‘segmentationprotocol_train_val.csv’, ‘2classprotocol_train_val.csv’, ‘3classprotocol_train_val.csv’, and ‘4classprotocol_train_val.csv’ for files related to semantic segmentation, 2-class, 3-class, and 4-class protocol, respectively. A similar notation has been followed in the ‘test’ folder. All CSV files contain image filenames and corresponding datasets, with the ChexPert dataset referred to as ‘chexpert’ and BIMCV-COVID19+ dataset referred to as ‘bimcv+’ in the ‘db’ column. CSV files corresponding to 2-class, 3-class, 4-class classification protocols contain 2, 3, and 4 columns, respectively, corresponding to their classes. If an image belongs to class *c*, the value for the column corresponding to *c* will be marked with a 1, while other columns are marked as 0 in the CSV file.
- Each CSV file in ‘train_val’ folder contains information regarding the training and validation split. The columns ‘split_1’ and ‘split_2’ specify which images are used for training and validation in a particular split. The images in the training split are marked with a ‘1’ and those in validation split with a ‘0’.

Information about data records for samples present in the BIMCV-COVID19+ dataset can be found in the preprint released with the dataset [18, 34]. The filenames specified for BIMCV-COVID19+ in the CSV files specify actual filenames of the images and not image paths in MIDS (Medical Imaging Data Structure) structure [38] as followed in the dataset. The filenames for CheXpert have been specified in the same format as those in ‘train.csv’ in the CheXpert-v1.0-small [26] dataset.

The CAAXR dataset is licensed under the CC BY-NC-SA license. The dataset is currently available only for non-commercial use. As specified in dataset licensing guidelines [39], *“The CC BY-NC-SA license allows reusers to distribute, remix, adapt, and build upon the material in any medium or format for noncommercial purposes only, and only so long as attribution is given to the creator.”* Proper maintenance of the dataset will be ensured at the mentioned weblinks. Since we are using publicly available CXR datasets, as per local ethical regulations, we do not require ethical clearance for the CAAXR dataset. The authors of the BIMCV-COVID-19+ dataset ensure identity anonymization of x-rays. They also ensure the confidentiality of demographic characters, relative identities, organizations involved, and the identity of any other personnel involved in the procedure. Both the BIMCV-COVID19+ and CheXpert datasets have been constructed ensuring that there is no personally identifiable data in the datasets [34, 37].

## Experimental design

As a part of this study, we introduce four protocols for evaluation. Three out of four protocols are designed for classification, and one for segmentation. A total of 3,288 samples are drawn from the BIMCV-COVID19+, and a total of 7,212 samples are drawn from the CheXpert database across all protocols. All protocols use 5-fold cross-validation. The data is split into an approximate 40-10-50 train, validation, and test split. The 50% data used for testing is consistent across the cross-validation experiments. The remaining 50% data is divided into train and validation sets. The algorithms are trained using only train and validation splits. This ensures a fair estimate of the model’s performance on the testing data. Data related to the protocol has been summarized in Table 2. Details of files used for training, validation, and testing from both datasets are provided in associated CSV files. The protocol CSV files along with trained baseline models for classification and segmentation can be downloaded from http://iab-rubric.org/caaxr2020.

**Table 2:** Proposed experimental protocol for the train, validation, and test set of the proposed annotation dataset. 2-fold cross-validation is performed. The source images for COVID-19 X-rays are taken from the BIMCV-COVID19+ database, while the rest of the images are from the CheXpert database. The corresponding disease abnormality is provided in this study.

S. No.	Protocol	Classes	Sub-Classes	Train		Test	
				Sub-class wise	Total	Sub-class wise	Total
D1	Disease Localization	Abnormal Regions	Lung specific abnormalities	-	828	-	828
		Other Regions	Healthy regions & other non-lung abnormalities				
C1	2-class classification	COVID	-	1188	2400	1200	2400
		Non COVID	Healthy	492		480	
			Non-COVID Pneumonia	240		240	
			Other Abnormalities	480		480	
C2	3-class classification	COVID	-	1188	3600	1200	3600
		Unhealthy Others	Non-COVID Pneumonia	480		480	
			Other Abnormalities	720		720	
		Healthy	-	1212		1200	
C3	4-class classification	COVID	-	1188	4800	1200	4800
		Non-COVID Pneumonia	-	1200		1200	
		Other Abnormalities	-	1200		1200	
		Healthy	-	1212		1200	

### Semantic segmentation protocol

For the semantic segmentation protocol, 1,656 samples from the BIMCV-COVID19+ dataset are used. There are two classes considered for semantic segmentation- unhealthy regions and other regions. The bounding box annotations for the samples are used to mark the unhealthy regions on the X-rays. The annotations for the radiological finding are drawn by creating a binary mask using the bounding box coordinates. These masked images are also resized to a resolution of 224 × 224. The 1656 annotated samples are randomly split into training and testing sets, with the testing set containing 828 samples. The details of the train, validation and test sets are provided in CSV files.

### Classification protocol

We define three classification protocols. The different classification protocols represent the various possible modes for COVID screening. The three protocols predict the chest X-ray images to be in one of two, three, or four classes, respectively. For instance, in the 2-class classification protocol, the primary focus is the presence or absence of COVID. Meanwhile, in the 3-class classification protocol, we demarcate a COVID X-ray from an otherwise unhealthy one.

- **2-class Classification (COVID/Non-COVID):** In the two-class classification protocol, images belong to one of the two classes- COVID or Non-COVID. 2,388 CXR images for COVID positive subjects are obtained from the BIMCV-COVID19+ dataset, while 2,412 Non-COVID infected CXR images are obtained from the CheXpert dataset. For maintaining balance and variance in the Non-COVID infected CXRs, X-ray images are either healthy, infected with non-COVID pneumonia, or have some other abnormalities. The testing set contains 2400 samples. It should be noted that the samples belonging to the training, validation, and testing splits in the segmentation protocol persist as samples in the training, validation, and testing splits of this protocol, respectively, thereby maintaining consistency across protocols.
- **3-class Classification (COVID/Non-COVID Unhealthy/Healthy):** In the three-class classification protocol, X-rays belong to one of the three classes- COVID, Non-COVID Unhealthy, or Healthy. The set of COVID 2,388 CXRs used in the previous classification protocol is used in this protocol. However, this time a subset of 4,812 X-rays is obtained from the CheXpert dataset for the Non-COVID Unhealthy and Healthy class samples. The samples in the Non-COVID Unhealthy class consist of CXRs with Non-COVID pneumonia and CXRs with other radiological abnormalities.
- **4-class Classification (COVID/Non-COVID Pneumonia/Unhealthy Other/Healthy):** In the four-class classification protocol, X-rays belong to one of the four classes- COVID, Non-COVID Pneumonia, Unhealthy Others, or Healthy. The set of 2,388 COVID chest X-rays used in all classification protocols is the same as the previous two protocols. A subset of 7,212 images is obtained from the CheXpert dataset for samples belonging to the Non-COVID Pneumonia, Unhealthy Others, and Healthy classes. The only negative result in the CheXpert dataset is “No finding”. This is treated as “Healthy” in our protocols. All other categories are considered as “Unhealthy”.

## Implementation details

We detail the steps involved in data pre-processing and data creation. Additional information has been provided to aid reproducibility of baseline results.

### Data pre-processing

This section details the pre-processing steps performed to perform experiments using deep models.

#### BIMCV-COVID19+ dataset

Images in the BIMCV-COVID19+ dataset are 16-bit grayscale high-resolution PNG images. Additionally, the metadata information specifies images that need to be contrast-inverted for proper viewing. The images are pre-processed by converting the PNG files into a standard 8-bit image format. Next, the key *“20500020”* is checked in the corresponding JSON metadata file for each image. This key corresponds to the Photometric Interpretation DICOM tag ((0028,0004)). If the key exists and its value is *“INVERSE”*, contrast-inversion is performed on the corresponding CXR image. All images are resized to a fixed resolution of 224 × 224 using bi-cubic interpolation.

#### CheXpert dataset

For the CheXpert dataset, the downsampled resolution CheXpert-v1.0 dataset is used. It has images of resolution 300 × 300 pixels in the PNG format. The images are pre-processed by resizing to a fixed resolution of 224 × 224 using bicubic interpolation.

### Data creation

The bounding box CSV file contains information of images from the BIMCV-COVID19+ dataset for which annotations are provided. The original dataset images can be downloaded from their website [18]. The files are pre-preprocessed as specified in the previous section. All pre-processing is performed using Python 3.6.9 on Linux, kernel 5.3.0-61-generic 18.04.1-Ubuntu SMP, architecture x86_64. Python libraries used for pre-processing include opencv-python (v. 4.2.0), pandas (v. 1.0.2), numpy (1.18.1), scikit-image (0.16.2), and json (2.0.9).

### Replicating experiments

This section provides implementation details and hyperparameter settings used for training the baseline models. The models are trained and saved after each epoch. The model giving minimum loss on the validation set is selected. This procedure is repeated for each cross-validation split. The models are then tested on the unseen test set. The hyperparameters for different segmentation and classification models are selected based on observing their performance on the validation sets.

#### Semantic segmentation

Training of UNet, SegNet and Mask-RCNN models is performed on Python 3.6.9 on Linux, kernel 5.3.0-61-generic 18.04.1-Ubuntu SMP, architecture x86_64. The models are trained using PyTorch (v.1.4.0) with the input size of images as 224 × 224 × 3. The training batch size is set as 4, and the model is trained for 25 epochs on the train and validation split using Adam optimizer with an initial learning rate of 0.0001. The Binary Cross-Entropy loss is minimized. Other Python library requirements include torchvision (v.0.5.0), tqdm (v.4.45.0), tensorboardX (v.1.1), and Pillow (7.0.0). The system with Nvidia RTX 2080Ti and Intel Xeon (having 48 cores) with 128 GB RAM is used for training the above models. For Mask-RCNN, implementation is done in PyTorch with input images of the same size. The training and validation batch size is set to 16, and the testing batch size is set to 8. The model is trained for 25 epochs, and, train and validation set with SGD optimizer with a learning rate of 0.001 and momentum 0.9. A combination of classification, localization, and segmentation mask loss are minimized where Binary Cross-Entropy loss is used for classification and segmentation task, and Smooth L1 loss for the classification task. For the aforementioned settings, one epoch for UNet, SegNet and Mask-RCNN models takes approximately 11, 12, and 55 seconds, respectively.

#### Classification

The classification models are trained by adding two fully connected dense layers of 1024 and 512 dimensions after the final convolutional layer of each model. During the experiments, convolutional layers of the models are frozen, and only the dense layers are updated using cross-entropy loss function. Models are trained for 30 epochs with Stochastic Gradient Descent (SGD) optimizer. The learning rate is set to 0.001 and momentum to 0.9. A batch size of 10 is used during training. The code is implemented in PyTorch (v1.4.0). All the experiments are performed on a system with Nvidia RTX 2080Ti GPU and Intel Xeon (having 48 cores) with 128 GB RAM. For the aforementioned settings, one epoch for DenseNet, ResNet, MobileNet and VGG takes approximately 8, 3, 4, and 10 seconds, respectively.

## Results and discussion

**Fig 4:**
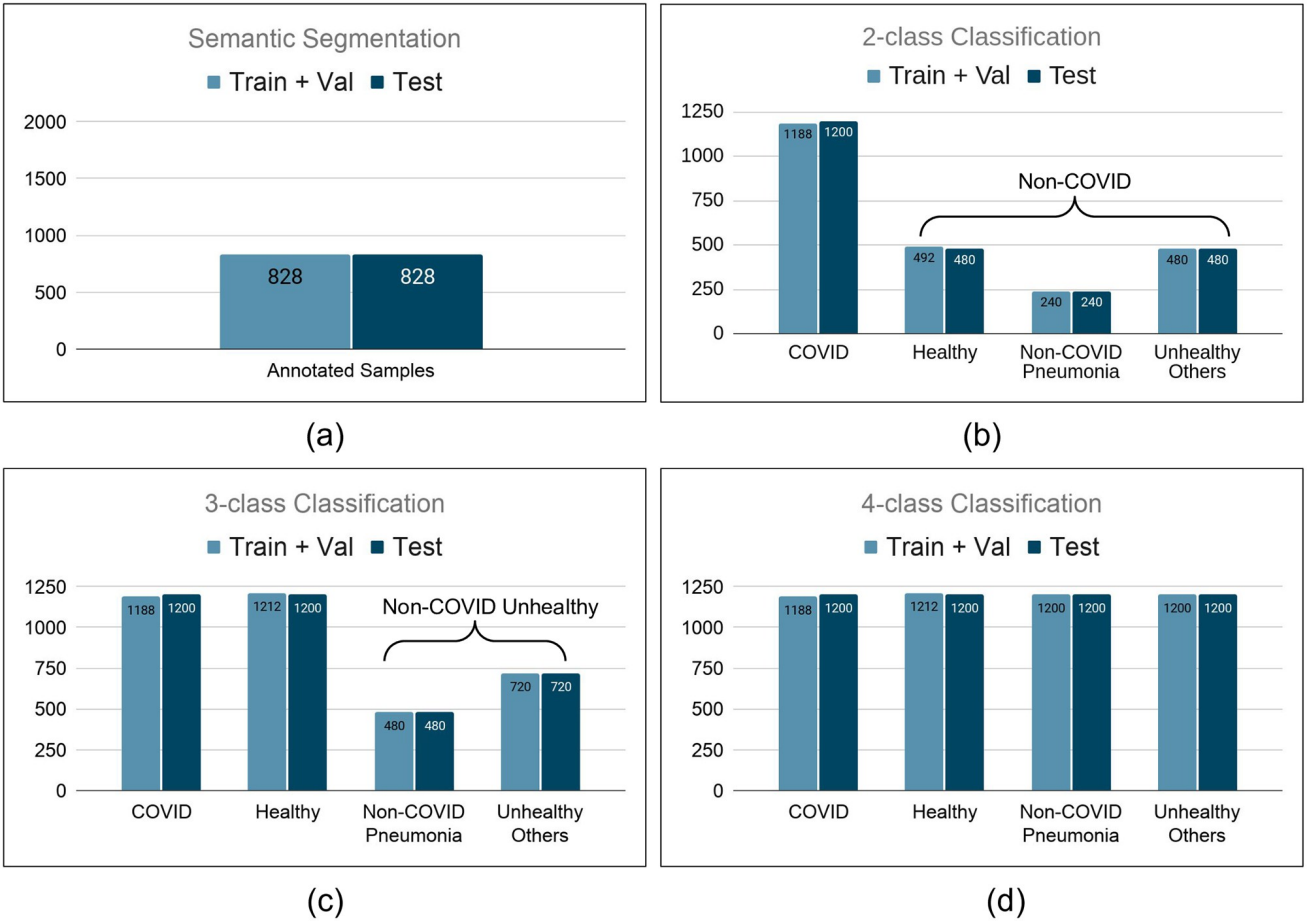
Number of samples present in protocol for (a) Semantic Segmentation. (b) 2-class Classification. (c) 3-class Classification. (d) 4-class Classification.

All experiments are performed using 2-fold cross-validation. The number of samples present in the train, validation, and test sets for all the protocols are shown in Fig 4.

### Semantic segmentation

As a part of this study, we provide baseline results for semantic segmentation using the described data protocols. We use three segmentation models, UNet [40], SegNet [41], and Mask-RCNN [42]. The performance is evaluated using two evaluation metrics, Intersection over Union (IoU) and DICE coefficient. Both metrics range from 0 to 1, measuring overlap between the ground truth and predicted samples. 1 denotes perfect and complete overlap, while 0 denotes a disjoint predicted mask and ground truth. IoU is the area of overlap between the predicted mask and the ground truth divided by the area of union between them. On the other hand, the DICE coefficient is calculated as two times the area of intersection of the predicted mask and ground truth, divided by the sum of their areas. The mean value of the respective metrics is calculated by averaging their values over the entire dataset.

**Fig 5:**
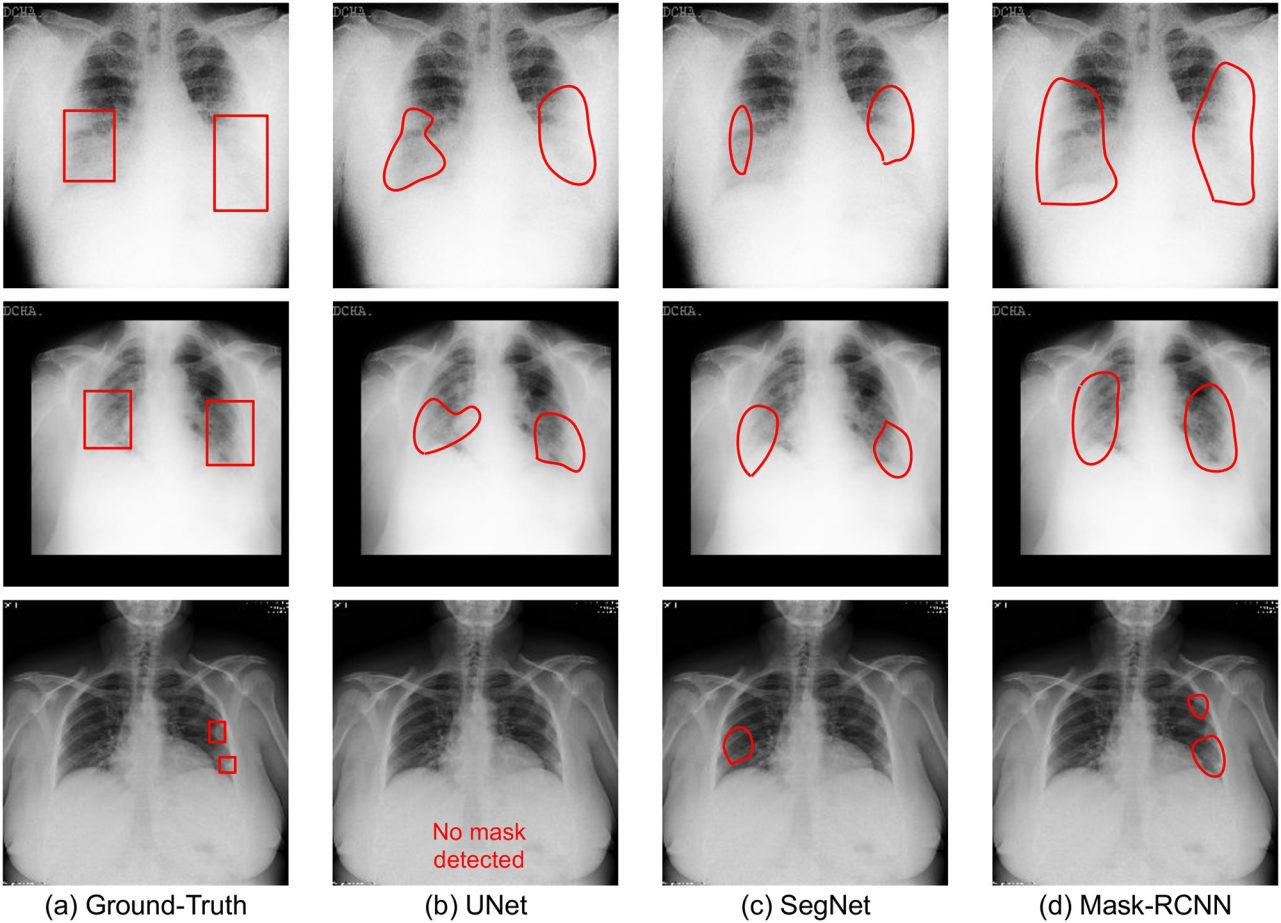
Samples of semantic disease segmentation for popular deep learning algorithms.

With new algorithms focusing on the quantification of pneumonia and tracking the progression of COVID-19 pneumonia, we further evaluate the models on a subset of the test set. This subset contains 768 X-rays with bounding box annotations for consolidation and ground-glass opacities only and provides a benchmark for pneumonia and other regions segmentation within the context of COVID-19. The results for segmentation on the test set and the aforementioned subset are shown in Table 3. We observe that Mask-RCNN performs the best among all the models with an IoU of 0.31 ± 0.01 and a DICE score of 0.50 ± 0.01 on the test set. This shows that Mask-RCNN can consistently learn the abnormalities present in the lungs better. Results for a few instances are shown in Fig 5. Mask-RCNN being the state-of-the-art algorithm in the arena of segmentation incorporates the concept of ROI Align, which brings a significant improvement in its performance over other techniques. UNet and SegNet lack this concept. We further observe that semantic segmentation models have difficulty localizing small affected regions in chest X-rays. This can be observed in Fig 3 where the affected regions are either missed or are predicted incorrectly.

**Table 3:** Evaluation of existing deep learning models for COVID-19 semantic segmentation.

Algorithm	Test Set		Consolidation Test Subset	
	IoU	DICE	IoU	DICE
UNet [40]	0.22 ± 0.03	0.32 ± 0.03	0.24 ± 0.03	0.35 ± 0.00
SegNet [41]	0.18 ± 0.01	0.28 ± 0.02	0.35 ± 0.03	0.30 ± 0.02
Mask-RCNN [42]	0.30 ± 0.02	0.43 ± 0.02	0.32 ± 0.02	0.46 ± 0.02

### Classification

In order to evaluate the classification performance, four deep learning models namely—DenseNet121 [43], MobileNetv2 [44], ResNet18 [45], and VGG19 [46] are trained separately using the five-fold cross-validation for each of the classification protocols. The results of the COVID classification are summarized in Table 4. The performance of the models is evaluated in terms of sensitivity at 99% and 90% specificity. We observe that MobileNetv2 yields the best results for 2-class and 3-class classification. Also, it achieves the best performance for 4-class classification at 99% specificity. Table 5 shows the class-wise classification accuracy for 2-class, 3-class, and 4-class classification using the four existing deep models. For 3-class and 4-class classification, the performance of the models for classifying other classes apart from the COVID class is low. This could be due to the small number of samples present in the dataset to learn the large diversity present in these classes. It is also observed that all the models achieve high performance for classifying the COVID class corresponding to all the protocols. Table 6 shows the Area under the ROC curve (AUC) for 2-class, 3-class, and 4-class classification using the four existing deep models. A similar observation of high performance for COVID class corresponding to all the protocols can also be drawn from Table 6. Further, Table 7 shows the results of significance testing using the t-test for the classification models. At a p-value < 0.05, the performance of MobileNetv2 and ResNet18 models for COVID class in 4-class classification is significantly different from DenseNet121. This observation is consistent with Table 5. For the models obtained using the 2-class and 3-class classification protocols, the differences in performance were found to be statistically insignificant.

**Table 4:** Evaluation of existing deep learning algorithms for COVID-19 prediction corresponding to all classification protocols. In the case of multi-class classification, a one-vs-all strategy is used to report sensitivity for COVID detection.

Algorithm	Sensitivity @ Y Specificity					
	2-class classification		3-class classification		4-class classification	
	Y = 99%	Y = 90%	Y = 99%	Y = 90%	Y = 99%	Y = 90%
DenseNet121 [43]	97.35 ± 0.06	99.52 ± 0.06	97.14 ± 0.16	99.57 ± 0.14	96.87 ± 0.30	99.52 ± 0.11
MobileNetv2 [44]	99.12 ± 0.11	99.75 ± 0.07	98.58 ± 0.17	99.57 ± 0.06	98.13 ± 0.21	99.62 ± 0.10
ResNet18 [45]	97.01 ± 0.41	99.70 ± 0.10	96.62 ± 0.40	99.60 ± 0.08	96.17 ± 0.55	99.43 ± 0.06
VGG19 [46]	94.77 ± 0.96	99.55 ± 0.28	93.84 ± 0.30	99.53 ± 0.11	94.02 ± 0.96	99.38 ± 0.12

**Table 5:** Class-wise classification accuracy corresponding to existing deep learning algorithms for all classification protocols.

Protocol	Classes	DenseNet121	MobileNetv2	ResNet18	VGG19
2-class classification	COVID	96.98 ± 1.22	98.81 ± 0.78	98.27 ± 0.32	96.74 ± 1.22
	Non-COVID	98.32 ± 0.65	98.68 ± 0.85	98.09 ± 0.30	97.45 ± 1.43
3-class classification	COVID	97.54 ± 1.04	97.92 ± 0.68	95.89 ± 1.35	96.43 ± 0.70
	Non-COVID Unhealthy	73.36 ± 4.34	74.73 ± 4.28	72.69 ± 1.37	72.06 ± 1.68
	Healthy	72.85 ± 4.04	72.79 ± 3.69	70.96 ± 1.43	71.71 ± 1.73
4-class classification	COVID	87.74 ± 4.70	97.27 ± 0.93	95.83 ± 1.59	90.81 ± 1.58
	Non-COVID Pneumonia	57.22 ± 10.89	57.63 ± 6.41	53.86 ± 3.24	56.06 ± 4.91
	Unhealthy Others	50.80 ± 3.21	50.54 ± 2.74	47.23 ± 2.13	51.20 ± 1.64
	Healthy	58.06 ± 8.61	57.03 ± 5.07	54.18 ± 5.53	55.14 ± 3.68

**Table 6:** Class-wise AUC corresponding to existing deep learning algorithms for all four classification protocols.

Protocol	Classes	DenseNet121	MobileNetv2	ResNet18	VGG19
2-class classification	COVID	0.998 ± 0.000	0.999 ± 0.000	0.998 ± 0.000	0.996 ± 0.001
	Non-COVID	0.998 ± 0.000	0.999 ± 0.000	0.998 ± 0.000	0.996 ± 0.001
3-class classification	COVID	0.998 ± 0.000	0.999 ± 0.000	0.998 ± 0.000	0.997 ± 0.000
	Non-COVID Unhealthy	0.895 ± 0.002	0.898 ± 0.001	0.889 ± 0.002	0.889 ± 0.003
	Healthy	0.894 ± 0.005	0.901 ± 0.001	0.889 ± 0.001	0.886 ± 0.003
4-class classification	COVID	0.998 ± 0.000	0.998 ± 0.000	0.997 ± 0.000	0.996 ± 0.000
	Non-COVID Pneumonia	0.820 ± 0.035	0.821 ± 0.040	0.795± 0.039	0.812 ± 0.032
	Unhealthy Others	0.788 ± 0.005	0.793 ± 0.004	0.768 ± 0.012	0.793 ± 0.006
	Healthy	0.830 ± 0.037	0.840 ± 0.037	0.822 ± 0.039	0.832 ± 0.028

**Table 7:** p-values obtained after performing t-test using the baseline models for the 4-class classification protocol.

	MobileNetv2	ResNet18	VGG19
DenseNet121	0.00004	0.00003	0.06119
MobileNetv2	-	0.99248	0.02548
VGG19	-	-	0.02375

The high performance of the COVID class could be due to the difference in the dataset properties as the COVID, and non-COVID samples are taken from two different datasets. This might include learning source properties of the X-ray machines or digital signatures introduced due to software-level image manipulations. For instance, BIMCV-COVID19+ dataset has CXR pixels converted from DICOM to nii.gz format and then to 16-bit PNG format after rescaling the dynamic range. This observation is strengthened by the fact the models have a subpar performance for classification within the classes from the CheXpert dataset, while the COVID-19 classification is consistently very good. Since the BIMCV-COVID19+ database contains COVID-19+ patient X-rays, it was essential to include X-rays that were healthy and/or affected by other abnormalities while developing an evaluation protocol for the dataset.

### Limitations

The annotations for CAAXR data constitute images from the BIMCV-COVID19+ database, which contains X-rays from COVID-19 positive patients. These X-rays are largely observed to be affected with consolidation/ground-glass opacities which are a characteristic of COVID-19 pneumonia. This leads to the annotations being skewed towards a particular abnormality. Further, the CAAXR protocols include CXR images from two different datbases, BIMCV-COVID19+ and CheXpert. We briefly discuss in the previous section how the high performance of the COVID classification could be due to the differences in the dataset properties of the COVID (BIMCV-COVID19+) and non-COVID (CheXpert) samples. In this direction, multiple studies have been performed which analyze the generalization abilities of deep learning models and how the models exploit confounding information [47]. These confounding factors might include learning source properties of the X-ray machines or digital signatures introduced due to software-level image manipulations. Further, CXRs of different datasets belong to different demography. The shift in demography can lead to distribution shifts that may affect the model performance [48]. For instance, the BIMCV-COVID19+ dataset contains patient data from Spain, specifically, the Valencian Region Medical Image Bank. As reported in the associated preprint [34], the age statistics of the database are consistent with those of the population of COVID-positive patients in Spain with a mean of approximately 63 years. The dataset contains CXRs corresponding to 45.92% females out of all the patients. On the other hand, while the complete CheXpert data [37] also contains approximately 45% females, images from the age group 45-64 are the most prominent. The CheXpert dataset contains studies from patients of Stanford Hospital. There is little that can be done to avoid the distribution shift between the COVID positive CXRs from one dataset and the COVID negative CXRs from another dataset. While this may introduce a source of bias, medical data is often obtained from different sources, and that should not affect a model’s performance. Therefore, it is of utmost importance that we address this bias while developing deep learning algorithms. We would also like to point out that even though we have baselined current deep learning models for all the protocols, we look forward to the community for improving these results in terms of performance, reliability, and explainability.

## Conclusions and broader impact

The pandemic has gravely hit the general population. With an aim to help the general populace and advance the usage of CXRs as a viable solution for efficient COVID-19 diagnostics, we have introduced the CAAXR database. We make the annotated data and protocols available to the scientific community. This large-scale data will be useful for machine learning and deep learning algorithms and can be used for learning radiological patterns observed in COVID-19 patients. The annotations will motivate the scientific community to evaluate for explainability in model predictions using the provided ground-truth annotations. Further, the protocols will facilitate ML and DL practitioners for unified large-scale evaluation of their algorithms.

While classification methods have provided human-level performance in some tasks [49], adding explainability without an expert in the loop is still an open problem in the medical domain. By adding annotation labels, explainability analysis can be done using semantic segmentation techniques like UNet, SegNet, and Mask-RCNN. The diseased/affected regions can be identified and localized through the output of the model. Further, these labels can be utilized for adding supervision to the models while training. Since the CAAXR dataset provides annotations for multiple CXR studies corresponding to a subject, the abnormality annotations can be used to extract spatiotemporal features [50]. These features can be applied for modeling disease growth in the lung region.
